# Superlubricity of pH-responsive hydrogels in extreme environments

**DOI:** 10.3389/fchem.2022.891519

**Published:** 2022-08-11

**Authors:** Allison L. Chau, Patrick T. Getty, Andrew R. Rhode, Christopher M. Bates, Craig J. Hawker, Angela A. Pitenis

**Affiliations:** ^1^ Materials Department, University of California, Santa Barbara, Santa Barbara, CA, United States; ^2^ Department of Chemistry and Biochemistry, University of California, Santa Barbara, Santa Barbara, CA, United States

**Keywords:** lubricity, tribology, friction, polyelectrolyte, swelling

## Abstract

Poly(acrylamide-*co*-acrylic acid) (P(AAm-*co*-AA)) hydrogels are highly tunable and pH-responsive materials frequently used in biomedical applications. The swelling behavior and mechanical properties of these gels have been extensively characterized and are thought to be controlled by the protonation state of the acrylic acid (AA) through the regulation of solution pH. However, their tribological properties have been underexplored. Here, we hypothesized that electrostatics and the protonation state of AA would drive the tribological properties of these polyelectrolyte gels. P(AAm-*co*-AA) hydrogels were prepared with constant acrylamide (AAm) concentration (33 wt%) and varying AA concentration to control the amount of ionizable groups in the gel. The monomer:crosslinker molar ratio (200:1) was kept constant. Hydrogel swelling, stiffness, and friction behavior were studied by systematically varying the acrylic acid (AA) concentration from 0–12 wt% and controlling solution pH (0.35, 7, 13.8) and ionic strength (*I* = 0 or 0.25 M). The stiffness and friction coefficient of bulk hydrogels were evaluated using a microtribometer and borosilicate glass probes as countersurfaces. The swelling behavior and elastic modulus of these polyelectrolyte hydrogels were highly sensitive to solution pH and poorly predicted the friction coefficient (*µ*), which decreased with increasing AA concentration. P(AAm-*co*-AA) hydrogels with the greatest AA concentrations (12 wt%) exhibited superlubricity (*µ* = 0.005 ± 0.001) when swollen in unbuffered, deionized water (pH = 7, *I* = 0 M) and 0.5 M NaOH (pH = 13.8, *I* = 0.25 M) (*µ* = 0.005 ± 0.002). Friction coefficients generally decreased with increasing AA and increasing solution pH. We postulate that tunable lubricity in P(AAm-*co*-AA) gels arises from changes in the protonation state of acrylic acid and electrostatic interactions between the probe and hydrogel surface.

## 1 Introduction

The term “superlubricity” was first coined by Hirano and Shinjo in 1993 while studying the friction between two mica sheets (Hirano and Shinjo, 1993). They theorized that the friction forces between two crystalline materials could be eliminated through the cancellation of opposing forces created by atomic lattice mismatches. Over time, the term “superlubricity” was adopted to describe the tribological behavior of any material system where the friction coefficient (*µ*) was less than 0.01 (Baykara et al., 2018; Martin and Erdemir, 2018; Wang et al., 2020). However, this value is not universally agreed upon. For soft materials, a more demanding threshold of *µ* ≤ 0.005 is often used to denote superlubricity (Pitenis et al., 2016; Liu et al., 2021), which will be adopted herein.

Aqueous systems with excellent lubricating properties are ubiquitous in biology, from articular cartilage that coats synovial joints (Sophia Fox et al., 2009) to mucin layers that act as a lubricating barrier for epithelial surfaces throughout the body (e.g., eyes, ears, reproductive tract, gastrointestinal tract, and respiratory tract) (Bansil and Turner, 2018; Wagner et al., 2018; Werlang et al., 2019). Hydrogels, a network of crosslinked hydrophilic polymer chains swollen in water, are often used as synthetic analogues of living tissue due to their comparable mechanical properties and inherent tunability. Since the first hydrogel was synthesized by Wichterle and Lím in their seminal work (Lím and Wichterle, 1958; Wichterle and Lím, 1960), there has been significant interest in potential tribological applications. Hydrogels are often used as anti-biofouling materials and coatings for biomedical devices that require low friction interfaces such as catheters (Yong et al., 2019). In many of these cases, polyelectrolyte and polyzwitterionic polymer brush systems are used to achieve the desired ultra-low friction coefficients (Chen et al., 19792009; Briscoe et al., 2006; Banquy et al., 2014; Yan et al., 2019). Researchers demonstrated that the length of surface-grafted polyelectrolyte polymer brushes can be varied to tune friction and achieve superlubricity (Ohsedo et al., 2004) while others synthesized a superlubricious double-network hydrogel by grafting polyelectrolyte polymer chains from the surface, obtaining friction coefficients in the range of 0.001–0.004 (Zhang et al., 2021). Rudy et al. (2017) covalently attached an entangled network of poly(acrylamide-*co-*acrylic acid) polymer chains to PDMS, achieving a friction coefficient of *µ* = 0.003 ± 0.005 in PBS solution. Superlubricity has also been demonstrated in bulk hydrogels, where the average spacing between neighboring polymer chains, termed mesh size (*ξ*), is a function of the molecular weight of the polymer chains, crosslinking density, and external factors such as pH, ionic concentration, and temperature (Caccavo et al., 2018). Bulk hydrogels with depth-wise mesh size gradients with *ξ* ≈ 50 nm at the surface have exhibited superlubricity (*μ* ≈ 0.001) in sliding contact with hydrogel probes (Pitenis et al., 2016). Wang et al. (2020) synthesized zwitterionic copolymer hydrogels that achieved superlubricity (*µ* ≈ 0.002) in water with sapphire countersurfaces and attributed the ultra-low frictional behavior to hydration lubrication caused by the formation of hydration layers surrounding the charges of the zwitterionic copolymers.

The structural tunability of hydrogels through temperature- or pH-induced swelling is another attractive quality, especially in the design of sensors (Johnson et al., 2004; Ying and Liu, 2021) and drug delivery capsules (Ravichandran et al., 1997; Ferreira et al., 2000). Poly(acrylic acid) (PAA) is a well-known pH-sensitive polyelectrolyte and is often copolymerized with other polymers such as poly(vinyl alcohol) (PVA) (Choi et al., 2012) or poly(acrylamide) (PAAm) (Lopez-Ureta et al., 2008; Jing et al., 2019) for greater mechanical strength. The effects of solution pH and ionic concentration on the swelling properties of PAA-based hydrogels have been thoroughly studied (Garces et al., 1994; Li et al., 2002; Zhou et al., 2003; Çaykara and Akçakaya, 2006; Sheikh et al., 2010; Thakur et al., 2011; Nesrinne and Djamel, 2017; Heidari et al., 2018; Mahon et al., 2019; Prouvé et al., 2021). Swelling increases when the pH of the solution exceeds the p*K*_a_ of PAA (≈4.7) due to the deprotonation of the carboxylic acid groups in acrylic acid (Sheikh et al., 2010). The formation of these negatively charged carboxylate ions (COO^−^) leads to electrostatic repulsion between polymer chains, which increases the hydrogel swelling capacity and mesh size (Zhou et al., 2003; Sheikh et al., 2010; Urueña et al., 2015). Conversely, when pH drops below the p*K*_a_ or if salt is added, swelling decreases (Li et al., 2002; Heidari et al., 2018; Mahon et al., 2019). This swelling, in turn, leads to changes in the stress relaxation behavior of the gel (Prouvé et al., 2021).

Notably, there have been few investigations studying the impact of solution pH on the tribological properties of bulk polyelectrolyte gels. Han et al. (2012) showed that thin (∼µm) multilayered devices composed of PAA and poly (allylamine hydrochloride) (PAH) swelled at low pH and exhibited lower friction as solution pH decreased. Ma et al. (2017) recently controlled the friction coefficient of PAA nanohydrogel brush systems by changing the solution pH *in situ*. Additionally, the pH range used to test the tunability of these polyelectrolyte systems is often limited between 1 and 12. To our knowledge, there have been no studies observing the effects of extremely acidic (pH < 1) or extremely basic (pH > 12) conditions on the mechanical characteristics of polyacrylamide-*co-*acrylic acid (P(AAm-*co*-AA)) hydrogels. Despite several in-depth studies examining the effects of pH on the swelling dynamics and mechanics of such polyelectrolyte gels, there are no systematic studies connecting solution pH and acrylic acid concentration to tribological properties. In this work, P(AAm-*co*-AA) hydrogels were synthesized with varying AA concentrations (0–12 wt%) and swollen in solutions of varying pH (0.35, 7, 13.8) and ionic strength (*I* = 0 or 0.25 M) with the goal of tuning the friction coefficient and achieving superlubricity. The changes in swelling and stiffness in response to extreme pH was also studied for these polyelectrolyte gels. Superlubricity was achieved in the P(AAm-*co*-AA) hydrogels with highest AA concentration (12 w%) in deionized water and NaOH.

## 2 Materials and methods

### 2.1 Hydrogel synthesis

**FIGURE 1:**
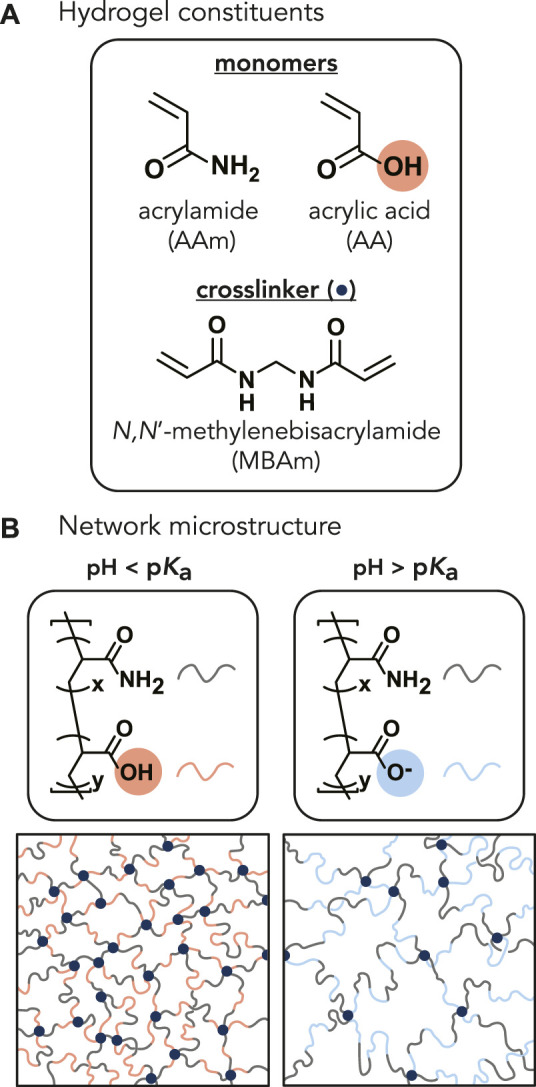
**(A)** Chemical structures of the monomers (acrylamide and acrylic acid) and crosslinker (*N*,*N*′-methylenebisacrylamide) (navy circle). The molar ratio of acrylamide to acrylic acid monomers varied from 1:0, 100:1, 20:1, 15:1, 10:1, and 7.5:1, corresponding to 0, 1, 5, 6, 9, and 12 wt% AA of the total monomer concentration. The monomer-to-crosslinker molar ratio of 200:1 was kept constant across all AA concentrations. **(B)** Representative P(AAm-*co*-AA) hydrogel network microstructures depicting the expected protonation state and interaction between polymer chains within the network as a function of solution pH. When the pH of the solution is less than the p*K*_a_ of the hydrogel, the acrylic acid should be protonated (denoted by red). When the pH > p*K*_a_, the acrylic acid should become deprotonated and negatively charged (denoted by blue), increasing electrostatic repulsion between polymer chains and increasing the mesh size.

Poly(acrylamide-*co*-acrylic acid) hydrogels (P(AAm-*co*-AA)) were synthesized via free radical polymerization. Stock solutions of acrylamide (AAm) (1 g/ml), *N*,*N*′-methylenebisacrylamide (MBAm) (25 mg/ml), ammonium persulfate (APS) (50 mg/ml), and *N*,*N*,*N*′,*N*′-tetramethylethylenediamine (TEMED) (50 mg/ml) were prepared in ultrapure water (Ω = 18.2 MΩ ⋅ cm). The final AAm concentration of all P(AAm-*co*-AA) hydrogels was 0.5 g/ml (corresponding to 33 wt%). The molar ratio of AAm:AA was controlled by adding uninhibited acrylic acid (AA) (neat) to the pre-polymerized solution. Six different molar ratios of AAm:AA (1:0, 100:1, 20:1, 15:1, 10:1, and 7.5:1) were synthesized, corresponding to 0, 1, 5, 6, 9, and 12 wt% AA of the total monomer concentration. All hydrogels will be referenced as P(AAm-*co*-AA)-*x*, where *x* represents the wt% of AA, or by the concentration of AA in wt%. The monomer:crosslinker molar ratio was maintained at 200:1 for all hydrogel samples (Figure 1). The pre-polymerized solutions were deposited between two flat polystyrene plates (surface roughness, *R*_a_ ≈ 20 nm) in ambient air and polymerized at room temperature (23°C) for a maximum of 2 h. Hydrogel sections (16 or 24 mm diameter) were equilibrated in either unbuffered deionized (DI) water (18.2 MΩ ⋅ cm) (pH = 7, *I* = 0 M), 0.5 M HCl (pH = 0.35, *I* = 0.25 M), or 0.5 M NaOH (pH = 13.8, *I* = 0.25 M). The hydrogels equilibrated in DI water for at least 72 h before testing while separate hydrogel samples equilibrated in either HCl or NaOH for at least 1 week (Supplementary Figure S1). The molarity of the NaOH solution was chosen to ensure complete (100%) neutralization (deprotonation) of all AA units in the hydrogel using the following formula (Eq. 1) (Arens et al., 2017):

$$DN=\;\frac{n\left(base\right)}{n\left(ionizable\;monomer\right)}\;\times 100\%$$ (1)

where *DN* is the degree of neutralization (%) and *n* is the number of moles. The same principle was applied when determining the molarity of the HCl solution to ensure complete protonation of all AA.

### 2.2 Swelling measurements

Hydrogels were equilibrated in unbuffered DI water (pH = 7, *I* = 0 M), 0.5 M HCl (pH = 0.35, *I* = 0.25 M), or 0.5 M NaOH (pH = 13.8, *I* = 0.25 M) for at least 4 weeks to ensure equilibrium swelling was reached. The gels were sectioned with a 6 mm diameter circular punch and then weighed (*m*_s_) using a Mettler Toledo XPR105DR analytical balance (repeatability ± 15 µg). Lens paper was used to gently wipe away excess liquid from the hydrogel surface before weighing. Samples were then dried in a vacuum oven for 10 days at 60°C and weighed to obtain their dry mass (*m*_d_). The water content (%) was calculated using Eq. 2 (Gong et al., 1999a). The reported water contents are average values and standard deviations from three samples unless otherwise stated.

$$water\;content\;\left(\%\right)=\frac{m_{\text{s}}-m_{\text{d}}}{m_{\text{s}}}\;\times \;100\%$$ (2)

### 2.3 Microindentation

**FIGURE 2:**
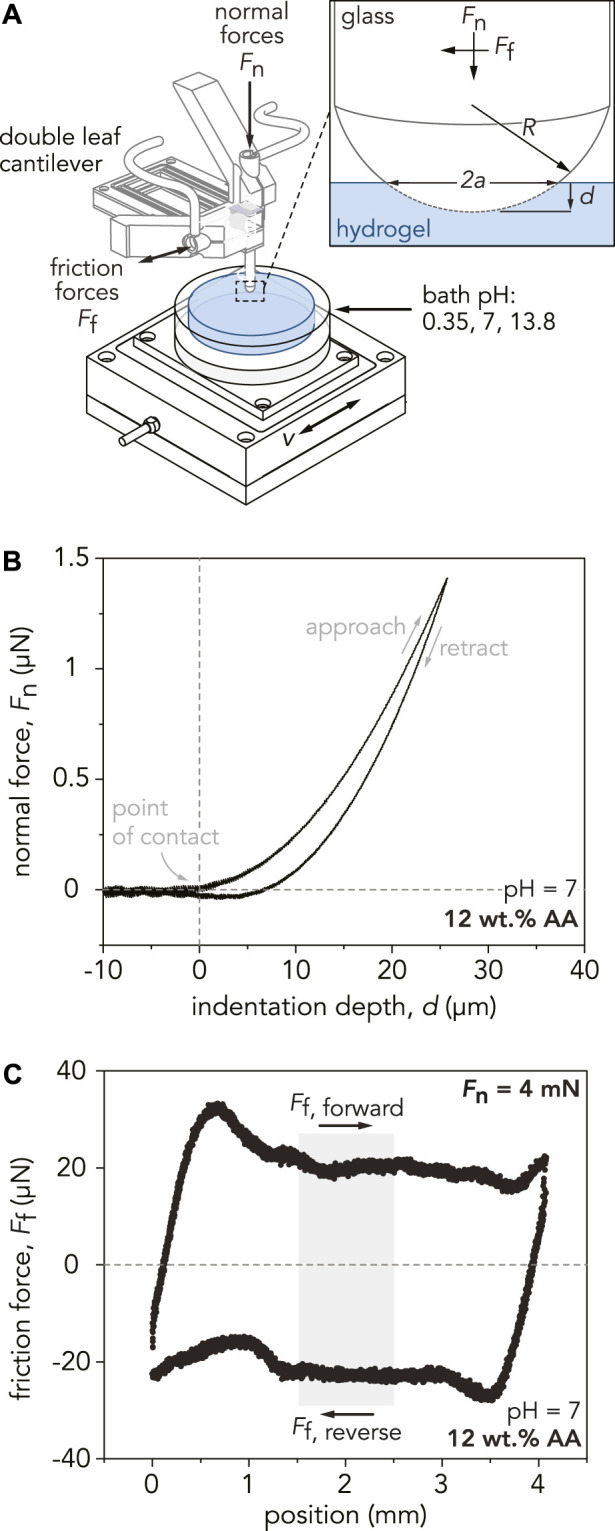
**(A)** Schematic depicting the custom-built linear reciprocating tribometer used for indentation and sliding experiment. The glass probe, with a radius of curvature *R*, is mounted to a double-leaf cantilever, which deflects in response to indentation and sliding. Capacitance probes measure the normal and tangential displacements of the cantilever, converting them into normal and friction forces. For indentation experiments, a defined normal force, *F*_n_, is applied to the sample at a specified indentation velocity, *v*_ind_. During sliding experiments, the sample stage displaces linearly at a defined sliding velocity, *v*, while applying a normal force, *F*_n_. The inset shows the area of contact between the probe and hydrogel, denoted by the contact area diameter, *2a*. The indentation depth of the probe into the gel is denoted by *d.* **(B)** Representative indentation curve for the P(AAm-*co*-AA)-12 hydrogel at pH = 7. A maximum applied normal force of *F*_n_ *=* 1.5 mN is applied to the hydrogel at an indentation velocity of *v*_ind_ = 10 µm/s. Using Hertzian contact mechanics, the reduced modulus, E*, is estimated by fitting the slope of the approach curve from the point of contact to *F*_n_ *=* 1 mN. **(C)** Representative friction force loop for the P(AAm-*co*-AA)-12 hydrogel at pH = 7 for one reciprocating cycle at an applied normal force of *F*_n_ = 4 mN and sliding velocity of *v* = 100 µm/s. Friction forces for each cycle are calculated by analyzing the middle 25% of the sliding path (light gray area). The friction coefficient of this individual cycle is *µ* = 0.005. To calculate the average friction coefficient for each hydrogel, the friction coefficients from 25 cycles are averaged.

Microindentation measurements were conducted with a custom-built linear reciprocating tribometer to determine the reduced elastic modulus, E*, of the hydrogels as shown schematically in Figure 2A. A hemispherical borosilicate glass probe was mounted to a double-leaf cantilever flexure with a normal spring constant of *K*_n_ = 150 µN/µm and tangential spring constant of *K*_f_ = 100 µN/µm. Borosilicate glass probes (radius of curvature, *R* = 3.1 mm) were used for gels swollen in DI water while probes with *R* = 2 mm were used for gels equilibrated in NaOH and HCl (Supplementary Figure S2). Hydrogel stiffness was evaluated over three different locations for each sample, and a maximum normal force of *F*_n_ = 1.5 mN (contact pressure *P* ≈ 10 kPa) was applied at a constant indentation velocity of *v*_ind_ = 10 µm/s. Five indentation measurements were performed at each location for a combined total of 15 indentations per sample. Experimental data were fit up to *F*_n_ *=* 1 mN using Hertzian contact mechanics theory, given by the expression in Eq. 3, by minimizing the sum of squared errors to solve for E* (Supplementary Figure S3)*.*

$$F_{\text{n}}=\;\frac{4}{3}E^{*}R^{1/2}d^{3/2}$$ (3)

In this equation, *R* is the probe radius of curvature, *d* is the indentation depth, and $E^{*}=\;E/\left(1-v^{2}\right)$ , where *E* is the compressive elastic modulus and v is the Poisson’s ratio of the hydrogel. Representative indentation curves are shown in Figure 2B and Supplementary Figure S4. The reported reduced elastic moduli are the averages and standard deviations of 45 total indentations spanning three separate gels.

### 2.4 Friction measurements

Tribological experiments were performed with a linear reciprocating tribometer, shown schematically in Figure 2A. Hydrogel friction coefficients were measured using a hemispherical borosilicate glass probe (radius of curvature, *R* = 2 or 3.1 mm). Samples were secured in a custom-built polyether ether ketone dish and submerged in unbuffered DI water (pH = 7, *I* = 0 M), 0.5 M HCl (pH = 0.35, *I* = 0.25 M), or 0.5 M NaOH (pH = 13.8, *I* = 0.25 M) for the duration of the experiment. Dishes were mounted to a motorized stage (Physik Instrumente, L-509.20DG10, 52 mm travel range) which provided linear reciprocating motion across a sliding path length, *l*, (1/2 cycle) of 4 mm to ensure the total sliding distance in one direction was at least eight times the estimated Hertzian contact area radius, *a*, at maximum normal load. A low sliding velocity of *v* = 100 µm/s was chosen to avoid approaching the soft elastohydrodynamic lubrication regime (Supplementary Section S4.1) (Hamrock and Dowson, 1978). The normal load (*F*_n_ = 4 mN) was maintained for at least 30 reciprocating cycles. Friction coefficients were calculated by averaging the normal and friction forces within the middle 25% of the sliding path. The following equation (Eq. 4) was used to calculate the average friction coefficient for each cycle, $\mu_{\text{cycle}}$ ,

$$\mu_{\text{cycle}}=\frac{\langle F_{\text{f},\text{forward}}\rangle -\;\langle F_{\text{f},\text{reverse}}\rangle }{2\langle F_{\text{n}}\rangle }$$ (4)

where $F_{\text{f},\text{forward}}$ is the average friction force in the forward direction and $F_{\text{f},\text{reverse}}$ is the average friction force in the reverse direction (Figure 2C). The average friction coefficient was determined by averaging *µ*_cycle_ over 25 cycles to obtain *µ*_sample_. The reported friction coefficients are the averages and standard deviations of *µ*_sample_ from three gels. The theoretical noise floor in friction coefficient measurements is *µ*_min_ = 0.000125 (Supplementary Section S4.2). Representative friction force loops for each pH condition can be found in Supplementary Figure S5.

## 3 Results and discussion

P(AAm-*co*-AA) hydrogels with varying AA concentrations (0–12 wt%) were placed in three solutions (0.5 M HCl, unbuffered DI water, 0.5 M NaOH) with pH (0.35, 7, 13.8) ranging below and above the p*K*_a_ of the copolymer hydrogel (p*K*_a_ ≈ 4.5) (Li et al., 2002) to alter the protonation state of the AA. We expected that all the AA was protonated while in HCl and deprotonated while in NaOH (Eq. 1, Supplementary Section S5). Since unbuffered DI water has no added counterions, the ionic strength is zero (I = 0 M). The water content, reduced elastic moduli (E*), and friction coefficients (*µ*) of the gels were compared as a function of AA concentration and pH. For all measurements, the averages and standard deviations were reported for three gels at each condition unless otherwise stated. Figure 1B schematically depicts the expected protonation state and interactions between polymer chains within the P(AAm-*co*-AA) hydrogel network as a function of the solution pH.

### 3.1 Swelling and pH

**FIGURE 3:**
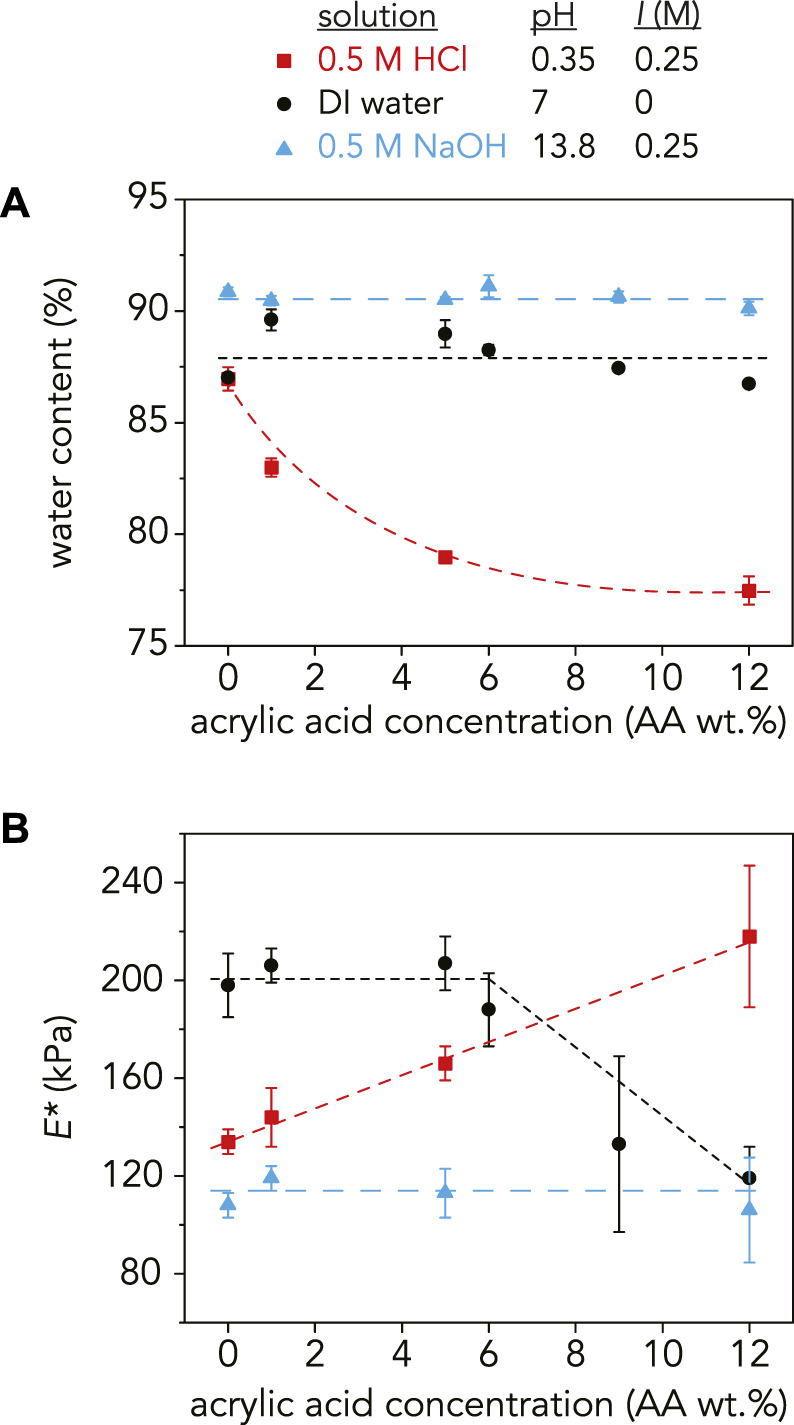
**(A)** Water content (%) and **(B)** reduced modulus, E*, as a function of AA concentration at pH = 0.35 (red squares), pH = 7 (black circles), and pH = 13.8 (blue triangles). At pH = 0.35, the water content monotonically decreased and E* increased with increasing AA as expected. In DI water, the swelling ratio increased with the addition of 1 wt% AA and then slightly decreased with increasing AA wt%. E* followed the same trend, decreasing at higher AA concentrations. At pH = 13.8, the water content and E* stays relatively constant with increasing AA concentration at 90–91% and 92–119 kPa, respectively. The dashed lines are guidelines and not meant to indicate a fit. For E*, each data point is an average of three samples (*n* = 3) with error bars as the standard deviation. For the water content measurements, *n* = 3 except for the following samples where *n* = 2:9 wt% AA in NaOH and 1 wt% AA, 6 wt% AA, 9 wt% AA, and 12 wt% AA in DI water.

Swelling in hydrogels is often measured with different metrics such as swelling percent (%) (Nesrinne and Djamel, 2017), fluid absorption capacity (Prouvé et al., 2021), and swelling ratio (Li et al., 2002). Water content, which represents the amount of solution absorbed by the gel in its fully swollen state (Gong et al., 1999a), was used to quantify swelling in our work (Figure 3A). Previous swelling studies of P(AAm-*co-*AA) hydrogels have demonstrated that swelling increases with increasing pH and acrylic acid concentration due to an increase in electrostatically repelling carboxylate ions caused by AA deprotonation (Garces et al., 1994; Li et al., 2002; Zhou et al., 2003; Thakur et al., 2011; Nesrinne and Djamel, 2017; Prouvé et al., 2021).

As expected, water content increased with increasing pH for all AA concentrations except for the P(AAm-*co-*AA)-0 hydrogel, where water content was 87% at both pH = 0.35 and pH = 7, indicating that in its fully swollen state, roughly 87% of the hydrogel mass was due to water while the remaining 13% was polymer. It is reasonable that the water content stayed constant for the P(AAm-*co-*AA)-0 hydrogels at these pH values because there were no ionizable carboxylic acid groups present in the gels. However, increasing AA concentration at pH > p*K*_a_ did not lead to an increase in water content as expected. The P(AAm-*co-*AA) hydrogels in DI water (pH = 7, *I* = 0 M) had a water content that fluctuated between 87–90% from 0 to 12 wt% AA. Similarly, when swollen in NaOH (pH = 13.8, *I* = 0.25 M), the water content stayed relatively constant between 90–91%. In contrast, the water content decreased monotonically from 87 ± 0.5% to 79 ± 0.2% as AA concentration increased for the hydrogels swollen in HCl (pH = 0.35, *I* = 0.25 M), which was expected due to greater amounts of hydrogen bonding between the protonated carboxylic acid and amide groups.

One possible reason for this discrepancy for the gels in NaOH was the presence of excess sodium counterions (Na^+^). Since the molarity of the NaOH solution was chosen to ensure complete deprotonation of all AA monomers, there was a stoichiometric excess of Na^+^ counterions in solution. Polyelectrolyte gels swell due to osmotic pressure from the counterions trapped within the network that ensure network electroneutrality (Zeldovich and Khokhlov, 1999; Zeynali and Rabbii, 2002). If there is an excess of counterions outside the network within the solution, this may reduce the osmotic pressure driving swelling. Additionally, the ionic strength of the solution has been shown to decrease swelling due to charge screening, reducing repulsion between polymer chains (Gong et al., 1999a; Li et al., 2002). One way to test this conjecture is to increase the ionic strength in DI water through the addition of a salt, such as NaCl, to observe any potential effects of charge screening on swelling. Another factor that may be influencing this AA concentration independence is crosslink degradation. Since MBAm is the crosslinker used in these P(AAm-*co*-AA) hydrogels, the amide is susceptible to attack in these extremely basic conditions. This could lead to the hydrolysis of the amide groups (Zeynali and Rabbii, 2002), decreasing the effective crosslinking density and forming additional acrylic acid groups (Supplementary Figures S6, S7). If crosslinks are breaking, this could overpower any differences caused by increasing AA concentration. One confirmation of this hypothesis is the increase in water content of the P(AAm-*co-*AA)-0 hydrogel at pH = 13.8 (91 ± 0.2%), despite the lack of AA.

For the hydrogels in DI water, the slight decrease in swelling with increasing AA concentration may be explained by an increase in total polymer concentration with the addition of AA (Supplementary Section S7, Supplementary Table S3 **)**. The total polymer concentration before swelling increased from 33 wt% for P(AAm-*co-*AA)-0 to 36 wt% for P(AAm-*co-*AA)-12. Therefore, changes in swelling due to increasing AA concentration may be masked by the changing initial polymer concentration.

### 3.2 Elastic modulus and pH 

The average reduced elastic modulus, E*, for the P(AAm-*co*-AA) hydrogels at each pH was obtained through microindentations along three different positions and averaged across three separate samples (Figure 3B). Prouvé et al. (2021) demonstrated that the elastic modulus of P(AAm-*co*-AA) hydrogels decreased with increasing AA concentration due to an increase in swelling. Therefore, the elastic modulus should be dependent on swelling and decrease with increasing water content.

For the P(AAm-*co*-AA) hydrogels at pH = 0.35, E* increased from 134 ± 5 kPa at 0 wt% AA to 218 ± 29 kPa at 12 wt% AA. This can be attributed to an increase in hydrogen bonding between carboxylic acid (-COOH) and amide (-CONH_2_) groups (Garces et al., 1994; Owens et al., 2007; Yang et al., 2010) due to a decrease in water content (87 ± 0.5% to 79 ± 0.2%). E* stayed relatively constant with increasing AA wt% (106 ± 21–119 ± 5 kPa) for the hydrogels at pH = 13.8, corresponding with their uniform water content (90–91%). However at pH = 7, E* was approximately 200 kPa for the P(AAm-*co*-AA) hydrogels with 0–6 wt% AA but decreased to E* = 133 ± 36 with 9 wt% AA. This deviates from the swelling results where water content increased slightly with 1 wt% AA and then monotonically decreased.

As expected, the gels in NaOH (pH = 13.8, *I* = 0.25 M) have a lower modulus than those in HCl (pH = 0.35, *I* = 0.25 M) for all AA concentrations due to greater swelling. Hence, it is perplexing that the gels in DI water (pH = 7, *I* = 0 M) exhibited the highest moduli at low AA concentrations, despite experiencing similar swelling behavior as the NaOH samples. One possible explanation is the gels in DI water swelled so much due to electrostatic repulsion between carboxylate ions that the chains extended and became rigid, which has been observed for various polyelectrolyte hydrogels (Okay and Durmaz, 2002; Horkay et al., 2006; Orakdogen and Boyaci, 2017a; Orakdogen and Boyaci, 2017b). However in all of these studies, the gels exhibited this non-Gaussian behavior and extensibility at higher ionic molar ratios of the charged species (>15 mol%) than those explored here. Additionally, we observe that E* decreases with higher AA wt% in DI water, so it is unlikely that chain stiffening due to electrostatic repulsion is occurring. Alternatively, others have hypothesized that effective crosslinking density decreases with increasing charge density for many polyelectrolyte hydrogels (Okay and Durmaz, 2002; Orakdogen and Boyaci, 2017b), which may explain why we observe a decrease in E* with increasing AA concentration. But if this were the case, the swelling results would have reflected this crosslinking density decrease by increasing in water content with AA wt%, which is not what was observed.

Another factor that may be influencing the elastic modulus of these gels is degradation caused by the extremely corrosive conditions of 0.5 M HCl (pH = 0.35) and 0.5 M NaOH (pH = 13.8). As previously mentioned, the crosslinker may be susceptible to hydrolysis at pH 13.8, decreasing the effective crosslinking density. Not only would this reduce the modulus compared to those in DI water, but it would also explain why there was not a significant change in E* with increasing AA wt% for the gels in NaOH. This hypothesis is plausible since it was observed that swelling significantly increased when the hydrogels equilibrated in basic conditions were placed in DI water (Supplementary Figure S7), implying that crosslinks may have broken during the initial swelling in NaOH.

### 3.3 Superlubricity in extreme environments

**FIGURE 4:**
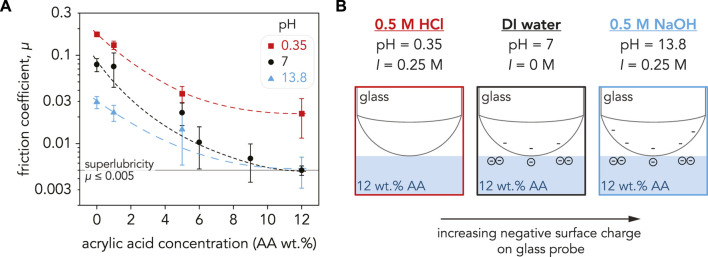
**(A)** Friction coefficient, *µ*, for the P(AAm-*co*-AA) hydrogels as a function of AA concentration plotted on a semi-log scale. For all pH values, the friction coefficient decreased with increasing AA concentration. Friction was the highest at pH = 0.35 (red squares) with 0 wt% AA (*µ* = 0.17 ± 0.009) and decreased to *µ* = 0.02 ± 0.01 at 12 wt% AA. The hydrogels with low AA concentration (0–5 wt% AA) had the lowest *µ* in NaOH (blue triangles), but *µ* was lower in DI water (black circles) at higher AA concentrations. Superlubricity was achieved for the P(AAm-*co*-AA)-12 hydrogel in DI water (*µ* = 0.005 ± 0.001) and NaOH (*µ* = 0.005 ± 0.002). The dashed lines are guidelines and not meant to indicate a fit. Each data point is an average of three samples (*n* = 3) with the error bars as the standard deviation. **(B)** Schematic depicting the surface charge of the glass probe at pH = 0.35, 7, and 13.8. Since the friction coefficient is a systems property and not an intrinsic material property, *µ* depends on the properties of the countersurface as well as the substrate. The surface charge of glass increases with increasing pH, and the electrostatic repulsion between the carboxylate ions in the P(AAm-*co*-AA) hydrogels and SiO^−^ ions in the glass probe may be contributing to the lower friction exhibited in DI water and NaOH.

The average friction coefficient for the P(AAm-*co*-AA) hydrogels were calculated over 25 sliding cycles at *F*_n_ = 4 mN. The effects of solution pH on the friction coefficients are shown in Figure 4A. By just altering the solution pH and AA concentration, the friction coefficients of the P(AAm-*co*-AA) hydrogels were tuned two orders of magnitude from *µ* = 0.17 ± 0.01 at pH = 0.35 (0 wt% AA) to achieving superlubricity (*µ* = 0.005 ± 0.001) at pH = 7 (12 wt% AA). In all pH conditions, the friction coefficients decreased with increasing AA concentration. The friction coefficients also decreased with increasing pH across all AA concentrations, except at 12 wt% AA. These results are within the range of values found by Ma et al. (2017), who observed tunable friction for pure polyacrylic acid nanohydrogel brushes. At pH = 2, their PAA brushes exhibited friction coefficients *µ* = 0.3–0.4, while low friction (*µ* < 0.01) was observed at pH = 12 (Ma et al., 2017). Dehghani et al. (2017) also demonstrated similar pH-tunability for polyelectrolyte brushes. However, we were able to demonstrate this tunability for a bulk hydrogel in both extreme and neutral pH conditions and achieved superlubricity with just 12 wt% AA. The effects of applied normal force and sliding velocity on the tribological properties of P(AAm-*co-*AA) hydrogels are described in Supplementary Section S8 **.**

For charge-neutral hydrogels, such as polyacrylamide, there is a strong correlation between the friction coefficient and mesh size, which is influenced by water content. Scaling concepts (de Gennes, 1979) and recent experimental investigations of charge-neutral aqueous gels (Pedro et al., 2021) suggest that the elastic modulus (*E*) decreases with increasing swelling due to an increase in the mesh size (ξ) where *E* ∼ *ξ* ^*−*3^; it is therefore expected that less swollen hydrogels have greater stiffness due to a smaller mesh size. Additionally, previous reports have demonstrated that the friction coefficient scales with the mesh size as *µ* ∼ *ξ* ^*−*1^ for PAAm hydrogels tested in a self-mated, Gemini configuration (where both the substrate and countersurface are composed of PAAm) (Urueña et al., 2015). However as demonstrated by the swelling, elastic modulus, and friction coefficient results herein, these scaling relationships do not extend to P(AAm-*co*-AA) hydrogels.

For charged polyelectrolyte hydrogels like P(AAm-*co*-AA), the correlation between water content and friction coefficient is not as clear. Liu et al. (2004) demonstrated that the friction coefficient of pure poly (acrylic acid) hydrogels decreased with increasing water content. Conversely, Gong et al. (1999a) postulated that the friction coefficient of strongly charged polyelectrolyte gels swollen in water has no dependence on water content. This is shown in our own results across all pH conditions, where there is no clear trend between E* or *µ* with water content (Supplementary Figures S9, S10). At pH = 0.35, *µ* decreased with increasing AA concentration despite the water content decreasing and stiffness increasing with AA wt%. Consequently, high water content and low elastic modulus do not necessarily indicate low friction, and other mechanisms need to be considered to understand the tribological behavior of P(AAm-*co*-AA) hydrogels.

### 3.4 Possible mechanisms for superlubricity

#### 3.4.1 Contributions of electrostatics to superlubricity

Electrostatic interactions have long been known to influence the tribological properties of polyelectrolyte hydrogels. Gong et al. (1999a) and others demonstrated that the friction coefficients of polyelectrolyte hydrogels depend on their charge density and the charge of the sliding countersurface (Oogaki et al., 2009; Ahmed et al., 2014). When the countersurface and hydrogel have the same charge, friction decreases with increasing surface charge density due to greater electrostatic repulsion between the two sliding interfaces and the formation of a solvent layer (Yoo et al., 1997; Gong et al., 1999a; Oogaki et al., 2009; Ahmed et al., 2014; Osaheni et al., 2020; Wang et al., 2020). Models have been developed to predict the thickness of this fluid layer, which depend on variables such as contact pressures and swelling ratios (Gong et al., 1999a; Sokoloff, 2010; Sokoloff, 2012; Sokoloff, 2013; Erbaş and Olvera De La Cruz, 2016; Tai et al., 2019).

For our experiments, borosilicate glass was used as the sliding countersurface (Figure 4B). Previous studies have demonstrated that glass has a negative surface charge density in water (Behrens and Grier, 2001) and that surface charge density increases with increasing pH (Shah et al., 1996; Gong et al., 1999b). Since friction is a systems property and is dependent on the countersurface material, having a negatively charged countersurface such as glass will lead to electrostatic interactions between the charged hydrogel surfaces (Osaheni et al., 2020). At pH = 0.35, neither the glass probe nor P(AAm-*co-*AA) hydrogel should have any charge, justifying the high friction coefficients. Conversely, at pH = 7 both the probe and hydrogel should be negatively charged. Repulsion between these sliding interfaces would explain the reduction in friction across all AA concentrations. Therefore, the superlubricity and low friction coefficients exhibited by P(AAm-*co*-AA) hydrogels in water may be attributed to greater electrostatic repulsion between the glass probe and hydrogel surface due to increasing negative surface charge with increasing AA concentration. The further reduction in friction at pH = 13.8 can be rationalized by the increase in negative surface charge on the glass probe due to the increase in pH. However, crosslink degradation may be the dominant driving force for the low friction coefficients exhibited by the P(AAm-*co*-AA) hydrogels in NaOH (Supplementary Figure S11). This would clarify why the P(AAm-*co*-AA) hydrogels with 0 wt% AA at pH = 13.8 possessed lower E* and *µ* than their counterparts at pH = 7 supposedly being pH-insensitive due to lack of ionizable functional groups (Turan and Çaykara, 2007; Martínez-Ruvalcaba et al., 2009). However, this still does not explain the behavior of P(AAm-*co-*AA) at pH = 0.35, which had the highest *µ* across all AA concentrations*.* Therefore, other mechanisms such as hydrogel microstructure may be contributing to the tribological behavior of these P(AAm-*co-*AA) hydrogels.

#### 3.4.2 Influence of hydrogel microstructure on superlubricity

As demonstrated herein, solution pH plays an important role in the swelling behavior, mechanics, and tribological properties of P(AAm-*co*-AA) hydrogels by altering the protonation state of AA. Conversely, the pH of the pre-polymerized solution significantly impacts the microstructure of copolymerized hydrogels. There have been many studies examining the effects of pH on the reactivity ratios of acrylamide and acrylic acid monomers (Cabaness et al., 1971; Rintoul and Wandrey, 2005; Riahinezhad et al., 2013; Ezenwajiaku and Hutchinson, 2021). While the actual values of these reactivity ratios are highly dependent on the reaction conditions (e.g., temperature, pH, ionic concentration, monomer concentration, etc.), the overarching trend is that the reactivity ratio of AAm (*r*_AAm_) increases while the reactivity ratio of AA (*r*_AA_) decreases with increasing pH (Cabaness et al., 1971; Rintoul and Wandrey, 2005; Riahinezhad et al., 2013).

**FIGURE 5:**
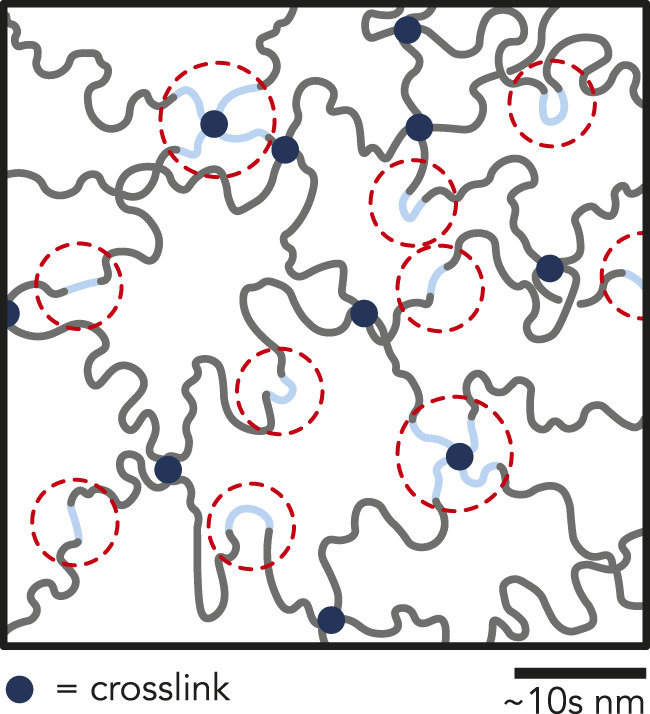
Illustration depicting possible clustering of AA (blue chains) within the P(AAm-*co-*AA)-12 hydrogel network. Due to low pH during polymerization, the reactivity ratio of acrylamide is less than unity, and the reactivity ratio of acrylic acid is greater than unity. Therefore, it is possible that AA-rich regions formed within the hydrogel network during polymerization leading to clusters of negatively charged domains, which may be partially responsible for superlubricity. Regions circled in red represent possible regions of counterion condensation and entrapment.

Since the pre-polymerized solution was not buffered, we observed that the pH decreased with increasing AA concentration (Supplementary Figure S12). Rintoul and Wandrey predicted that *r*_AAm_ *<* 1 and *r*_AA_ > 1 for the pH range of the pre-polymerized solution (pH = 2.7–3.6) (Rintoul and Wandrey, 2005). Because the reactivity ratio of AA is greater than unity, there may have been preferential clustering of AA during copolymerization. Therefore, these P(AAm-*co*-AA) hydrogels likely do not have a homogeneous distribution of AA monomers throughout the network and instead may contain microscale regions of high AA concentration, which could lead to heterogeneous charge distributions within the hydrogel (Figure 5). Others have noted microstructural inhomogeneities even in homopolymerized hydrogels (Gombert et al., 2020). Zeldovich and Khokhlov (1999) developed a model for inhomogeneous polyelectrolyte hydrogels with a heterogeneous distribution of charges. They posited that this inhomogeneous clustering of charge leads to counterion entrapment, prevents the counterions from contributing to the osmotic pressure and becoming “osmotically passive”. If clusters of AA regions within the P(AAm-*co*-AA) hydrogel formed, counterions may have gathered around these AA pockets and become osmotically passive, possibly clarifying the swelling behavior of the gels in NaOH. Recent investigations of P(AAm-*co-*AA) hydrogels with 8 wt% total monomer concentration and > 20 wt% AA demonstrated that AA-rich and AAm-rich domains formed under confinement, further supporting this hypothesis (Deptula et al., 2022). The potential for AA clusters to exist in the network presents opportunities for future investigations using advanced microscopic and spectroscopic techniques. The contributions of potential AA cluster formation on the tribological properties of P(AAm-*co*-AA) hydrogels remain to be established, yet we postulate that negative charge clustering may lead to local repulsion between the hydrogel surface and the glass probe and interesting tribological properties that require further exploration.

#### 3.4.3 Hydration lubrication often leads to superlubricity

For charged systems, hydration lubrication is often cited as a potential mechanism for the observed low friction. Hydration lubrication is a lubrication regime that can occur between charged, aqueous systems due to the formation of hydration layers surrounding the charges (Raviv et al., 2003; Gaisinskaya et al., 2012; Klein, 2013; Wang et al., 2020; Lin and Klein, 2022). While the water molecules within the hydration layer are strongly attached to their charges, they are also dynamic, with the dipoles constantly fluctuating and the water molecules within the hydration shell rapidly exchanging with those within the bulk. Due to this mobility, these hydration shells fluidly shear when the applied shear rates are less than the relaxation rates of the hydration shells (Gaisinskaya et al., 2012; Klein, 2013). Coupled with the repulsion that occurs between hydration layers when in contact, hydration lubrication leads to extremely low friction coefficients. Counterions can also play a role in friction reduction due to the short-range repulsion of their own hydration shells.

Many of the studies observing hydration lubrication were accomplished with mica substrates, which are molecularly smooth, coated with charged polymer brushes under confinement. When the separation distance between two charged polymer brush layers is smaller than the radius of gyration of the polymer chains, shear occurs across the hydration layers rather than the brush interface, reducing friction (Raviv et al., 2003; Klein, 2013). A more recent paper by Wang et al. (2020) also attributed the superlubricity of their bulk polyelectrolyte hydrogel to hydration layers.

However, it has also been shown that anions are not as hydrated as cations and are not as effective at providing hydration lubrication (Klein, 2013). Additionally, studies of hyaluronan–aggrecan complexes that possess COO^−^ groups and OSO_3_^−^ groups have also shown that they have weak hydration layers that are not strongly bound and are not efficient at hydration lubrication (Seror et al., 2012). Similarly, the P(AAm-*co-*AA) hydrogels have COO^−^ charged groups. Therefore, it is plausible that hydration lubrication may be one aspect contributing to the superlubricity demonstrated in our P(AAm-*co-*AA) hydrogels but not the only component.

## 4 Concluding remarks

We demonstrated that poly(acrylamide-*co*-acrylic acid) (P(AAm-*co*-AA)) hydrogels achieved superlubricity by tuning the acrylic acid (AA) concentration (0–12 wt%), solution pH (0.35, 7, 13.8), and ionic strength (*I* = 0 or 0.25 M). The swelling behavior and mechanical and tribological properties of these P(AAm-*co*-AA) hydrogels were characterized.

In 0.5 M HCl (pH = 0.35, *I* = 0.25 M), the carboxylic acid groups remain fully protonated, and the gels had the lowest water content and highest friction coefficients across all AA concentrations due to hydrogen bonding between carboxylic acid and amide groups. In 0.5 M NaOH (pH = 13.8, *I* = 0.25 M), the carboxylic acids groups are fully deprotonated, and the gels had the highest water content, lowest moduli, and lowest friction coefficients with 0–5 wt% AA. This molarity of NaOH was chosen to ensure complete deprotonation of the AA, but extremely basic conditions may have had adverse effects on crosslinking concentration through the hydrolysis of amides in the crosslinker. Hydrogels equilibrated in unbuffered DI water exhibited the highest moduli despite having high water content. The friction coefficient generally decreased with increasing AA and increasing solution pH, as expected. Superlubricity was achieved with 12 wt% AA in DI water and NaOH. The pH of the pre-polymerized solution may affect the microstructure of the P(AAm-*co*-AA) hydrogels, and AA clusters may form and induce inhomogeneous charge distributions. The contribution of this inhomogeneity to the tribological properties has yet to be explored, but we postulate that tunable lubricity arises from changes in the protonation state of acrylic acid and electrostatic interactions between the glass probe and hydrogel surface.

## Data Availability

The original data and contributions presented in this manuscript are included within the article/Supplementary Material. Please direct inquiries to the corresponding author.
