# Supplementary material for: Superlubricity of pH-responsive hydrogels in extreme environments
Source: Front Chem. 2022 Aug 11;10:891519. doi: 10.3389/fchem.2022.891519 (PMC9405656; doi:10.3389/fchem.2022.891519)
Supplement: Supplementary file 1 [file DataSheet1.PDF]

Supplementary Material

1 Swelling Measurements

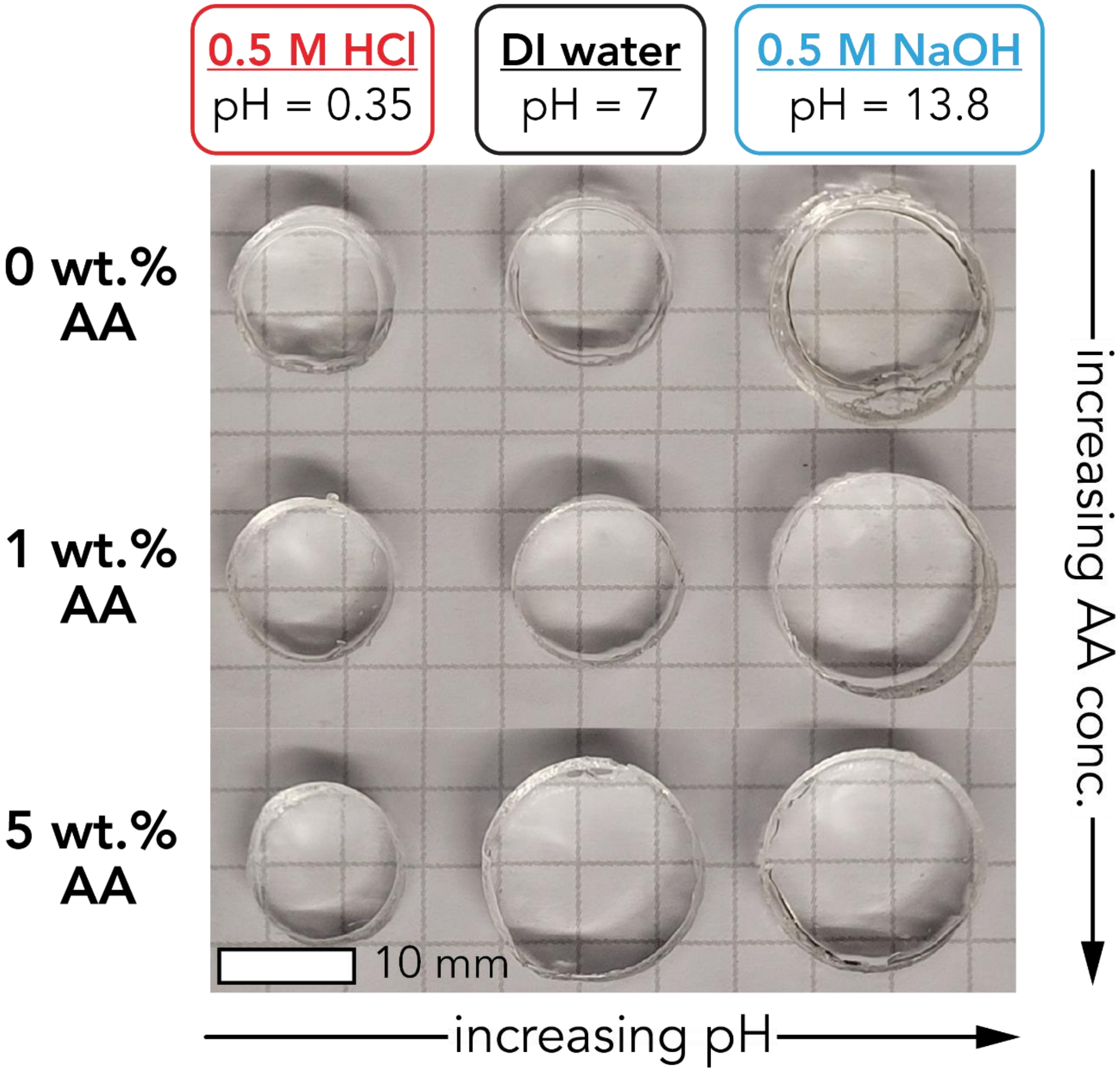

Fig S1. Comparison of P(AAm-*co*-AA) hydrogels with varying AA concentrations swollen in 0.5 M HCl (pH = 0.35, I = 0.25 M), unbuffered DI water (pH = 7, I = 0 M), or 0.5 M NaOH (pH = 13.8, I = 0.25 M) after reaching equilibrium swelling (4 days). Before swelling, all hydrogels had a diameter of 10 mm. Scale bar: 10 mm.

2 Optical Profilometry of Glass Probes

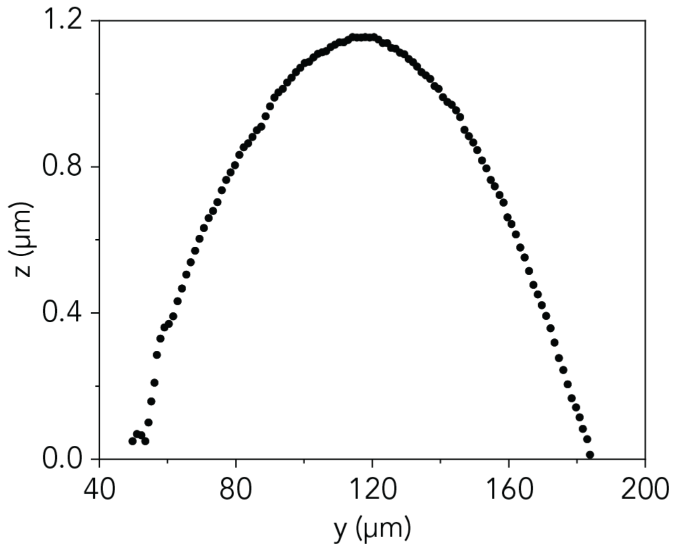

Fig S2. Optical profilometer trace of the hemispherical glass probe with radius of curvature R = 2 mm used during microindentation and friction experiments for the hydrogels in NaOH and HCl.

3 Microindentation Curves

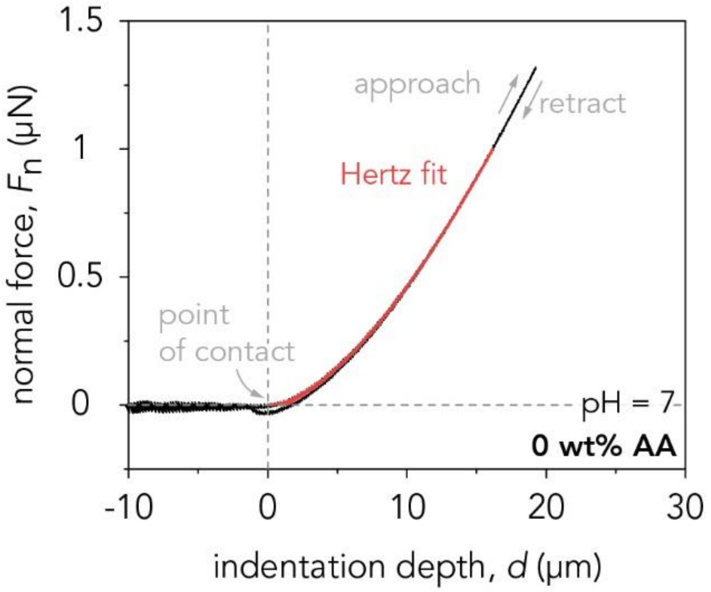

Fig S3. Microindentation curve for the P(AAm-*co*-AA)-0 hydrogel swollen in DI water with the approach and retraction parts of the curve labeled along with the point of contact. The Hertzian contact mechanics model (red) is fit to the approach curve up to F = 1 mN.

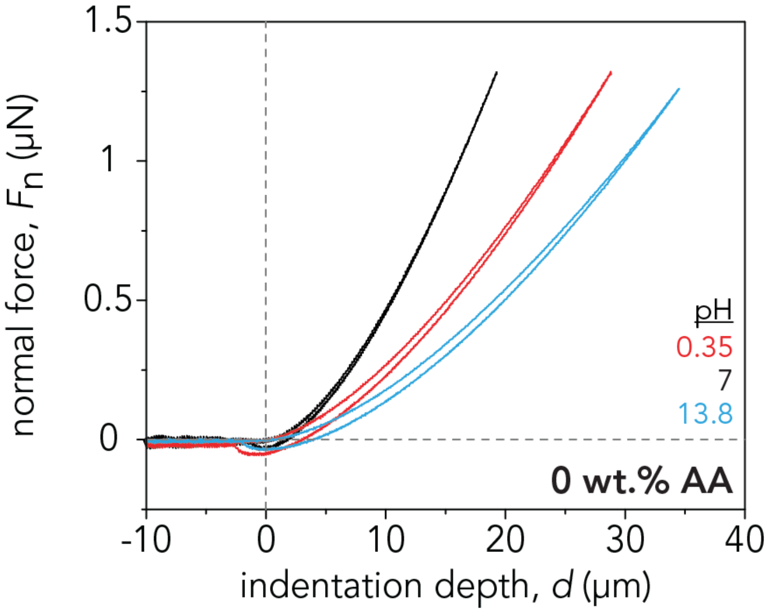

Fig S4. Representative indentation curves comparing the P(AAm-*co*-AA)-0 hydrogels at pH = 0.35 (red), pH = 7 (black), and pH = 13.8 (blue). There is not a monotonic decrease in the modulus with increasing pH.

4 Friction Measurements

4.1 Minimum Film Thickness Calculations

The Sommerfeld number, S, is a dimensionless parameter that is proportional to the viscosity of the lubricating fluid, the sliding velocity, v, and the applied normal force, F_n, as $S \; \alpha \; \frac{\eta v}{F_n}$.(1) The Stribeck curve plots the friction coefficient as a function of the Sommerfeld number for two sliding interfaces and partitions it into four main lubrication regimes: boundary, mixed, elastohydrodynamic lubrication (EHL), and hydrodynamic lubrication. Boundary lubrication occurs when the two sliding interfaces are in direct contact with each other whereas EHL and hydrodynamic lubrication occurs when the fluid film is thick enough to separate the two sliding interfaces. Due to this lubricating fluid layer, the friction coefficients measured within this regime are lower than those in boundary lubrication. The lubrication mode is highly dependent on applied force and sliding speed, with boundary lubrication typically occurring at lower sliding speeds and higher applied forces. To ensure that the friction coefficients were measured within the boundary lubrication regime rather than the EHL regime, the sliding speed of v = 100 μm/s and normal force of F_n = 4 mN were carefully chosen. Using soft-elastohydrodynamic lubrication theory developed by Hamrock and Dowson, (2) the minimum fluid film thickness, h_{min}, for soft-EHL lubrication was estimated between 9 – 17 nm, which was lower than the combined surface roughness of the probe and hydrogel surface, using **Eqn. S1**.

$$h_{min} = 2.8R^{0.77}(\eta_o v)^{0.65}E'^{-0.44}F_n^{-0.21} \qquad \text{Eqn. S1}$$

where R is the probe radius of curvature, η_o is the viscosity of the fluid, v is the sliding velocity, F_n is the applied normal load, and $E' = 2E^*$ where E^* is the reduced elastic modulus of the sample. The minimum fluid film thickness was calculated by using the viscosity of water (η_o = 8.9 x 10^{-4-} Pa·s), v = 100 µm/s, F_n = 1 and 4 mN, and E^* = 100 and 240 kPa (the minimum and maximum measured E^* for the P(AAm-*co*-AA) hydrogels). **Table S1** displays the resulting h_{min} values.

Table S1. Minimum fluid film thickness values for the P(AAm-*co*-AA) hydrogels with the lowest and highest reduced elastic moduli at the lowest and highest applied normal force.

	***E** = 100 kPa**	***E** = 240 kPa**
**F_n = 1 mN**	17 nm	12 nm
**F_n = 4 mN**	13 nm	9 nm

Since h_{min} was less than the estimated surface roughness of the hydrogels ($R_a \approx$ 20 nm), there was likely contact between the hydrogel surface and probe, indicating boundary-like lubrication. If h_{min} was significantly greater than the surface roughness of the hydrogel, then the measured superlubricity would be more likely due to a thick fluid film layer rather than the chemical composition of the hydrogel surface. At an applied normal force of 4 mN, the maximum pressure during sliding ranged between 9 – 15 kPa, depending on E^* of the P(AAm-*co*-AA) hydrogel. Since E^* scales with osmotic pressure and the applied contact pressures are much lower than E^*, it is unlikely that fluid flow or draining occurred, suggesting that the friction coefficients measured are not due to fluid flow or soft-EHL (3).

The track length was chosen so the distance was at least 4 times the contact area diameter to ensure that the probe moved out of its initial contact area zone when sliding. **Table S2** shows the minimum track length necessary for applied normal forces of 1 mN and 4 mN for hydrogels with reduced elastic moduli of 100 kPa and 240 kPa. Hertzian contact mechanics was used to estimate the contact area diameter, *d* (**Eqn. S2**).

$$d = 2\left(\frac{3F_n R}{4E^*}\right)^{1/3} \qquad \text{Eqn. S2}$$

Table S2. Minimum track length required to ensure that the sliding path is at least four times the contact area diameter, assuming Hertzian contact mechanics.

	***E** = 100 kPa**	***E** = 240 kPa**
**F_n = 1 mN**	2.3 mm	1.7 mm
**F_n = 4 mN**	3.6 mm	2.7 mm

4.2 Noise Floor Calculations for Tribometer

The noise floor, or minimum detectable friction coefficient, for the tribometer was estimated with the following equation:

$$\mu = \frac{F_\text{f}}{F_\text{n}} = \frac{K_\text{f} \cdot x}{F_\text{n}} \qquad \text{Eqn. S3}$$

where K_f is the tangential spring constant of the double-leaf cantilever, x is the minimum detectable displacement by the capacitance probes, and F_n is the applied normal force. For our experiments, K_f = 100 μN/μm. F_n = 4 mN, and the capacitance probes (Lion Precision, C5R-0.80-2.0) used to measure the displacement have a 5 nm resolution (x = 5 nm).

4.3 Friction Loops

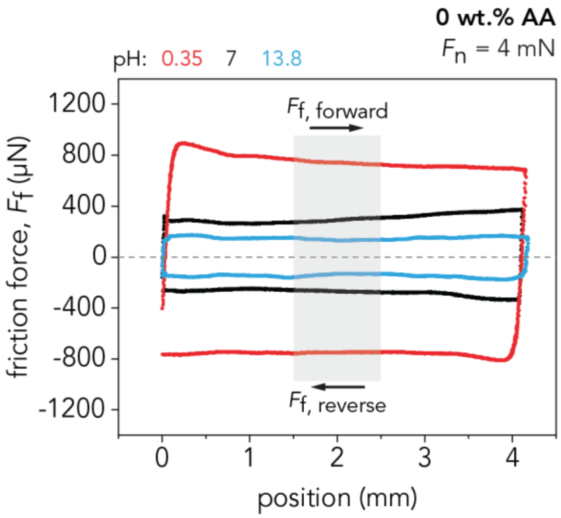

Fig S5. Comparison of representative friction force loops as a function of pH for the P(AAm-*co*-AA)-0 hydrogels. The forward and reverse directions of the friction loop are labeled. The middle 25% of the loop (gray area) is used to calculate the friction coefficient. As pH increases, the friction force decreases.

5 Henderson-Hasselbalch Equation

Rearranging the Henderson-Hasselbalch equation, the ratio of deprotonated acrylic acid (A^-) to protonated acrylic acid (HA) is as follows:

$$R = \frac{[A^-]}{[HA]} = 10^{(pH - \text{p}K_\text{a})} \qquad \text{Eqn. S4}$$

The fraction of protonated AA can be estimated as f_{HA} = 1/(1+R) while the fraction of deprotonated AA can be estimated as f_{A^-} = R/(1+R). With a pK_a = 4.5, f_{HA} = 0 and f_{A^-} = 1 when pH = 13.8 (0.5 M NaOH). When pH = 0.35 (0.5 M HCl), f_{HA} = 1 and f_{A^-} = 0. Therefore, it is expected that the AA is fully protonated in the HCl solution and deprotonated in the NaOH solution.

6 Degradation of Crosslinks

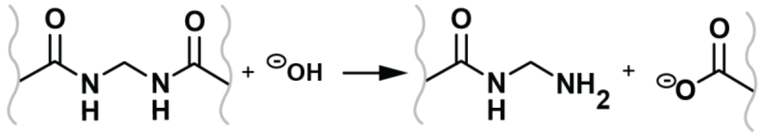

Fig S6. Possible hydrolysis of *N,N'*-methylenebisacrylamide leading to crosslink degradation within the hydrogel network.

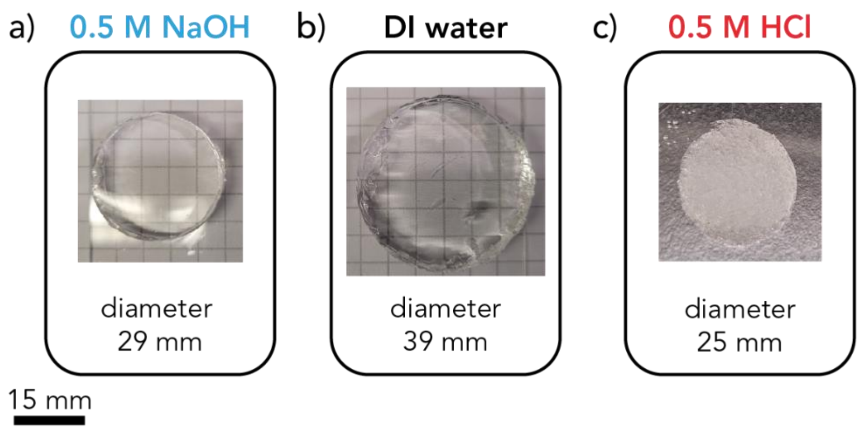

Fig S7. Comparison of the P(AAm-*co*-AA)-9 hydrogels swollen in various solutions. Scale bar: 15 mm **(a)** When placed in 0.5 M NaOH right after polymerization, the hydrogel reached equilibrium swelling. In this case, the hydrogel swelled to a diameter of 29 mm from a starting diameter of 16 mm. **(b)** When transferred directly from the NaOH solution into pure DI water, the hydrogel swelled to a diameter of 39 mm after three days, indicating that crosslinks might have been potentially broken while swelling in NaOH. **(c)** Within hours of being transferred from DI water into a 0.5 M HCl solution, the hydrogel collapsed and became brittle.

7 Hydrogel Synthesis

Table S3 Mass of each component in the pre-polymerized hydrogel solutions, where T represents the total polymer content and C represents the crosslinker concentration.

**AA:AAm (molar ratio)**	**AA/AAm molar ratio**	**T (wt%)**	**C (wt%)**	**AAm (mg)**	**AA (mg)**	**MBAm (mg)**	**TEMED (mg)**	**APS (mg)**	**MilliQ water (mg)**
0	0/100	33.4	1.1	2500	0	27.1	20.4	20.1	5000
0.01	1/99	33.6	1.1	2500	25.3	27.4	20.4	20.1	5000
0.02	2/98	33.8	1.1	2500	50.7	27.7	20.4	20.1	5000
0.05	5/95	34.5	1.1	2500	126.7	28.5	20.4	20.1	5000
0.07	6/94	34.9	1.1	2500	169	28.9	20.4	20.1	5000
0.10	9/91	35.6	1.1	2500	253.4	29.8	20.4	20.1	5000
0.13	12/88	36.3	1.1	2500	337.9	30.7	20.4	20.1	5000

The total polymer content (T, wt%) and crosslinker concentration (C, wt%) were calculated with **Eqn. S5 and S6**.(4,5)

$$T\ (w/w)\% = \frac{AAm\ (g) + AA\ (g) + MBAm\ (g)}{total\ mass\ (g)} x100 \qquad \text{Eqn. S5}$$

$$C\ (w/w)\% = \frac{MBAm\ (g)}{AAm\ (g) + AA\ (g) + MBAm\ (g)} x100 \qquad \text{Eqn. S6}$$

8 Effects of Sliding Speed and Normal Force on Friction Coefficients

It has been demonstrated by Gong and others that the friction coefficient for charge-neutral and polyelectrolyte hydrogels is highly dependent on the testing conditions, including sliding speed and applied normal load (6–9). Urueña *et al.* demonstrated that the friction coefficient of charge-neutral polyacrylamide hydrogels decreased with increasing applied normal force (7) while Gong *et al.* corroborated this trend for several other charge-neutral and polyelectrolyte hydrogels (8). Similar results were found for copolymerized zwitterionic hydrogels within the hydrodynamic lubrication regime (9). For the P(AAm-*co*-AA) hydrogels, the effects of applied force on the friction coefficient varied depending on AA concentration and pH (**Fig. S8)**. At pH = 13.8 and lower AA concentrations (0 – 5 wt.%), the friction coefficient decreased with increasing force. But for the gels at lower pH (pH = 0.35 and 7) across all AA concentrations and for the gels at pH = 13.8 at 12 wt%, the friction coefficient stayed relatively constant with increasing force. The friction coefficient of hydrogels also has a dependence on sliding speed and can either increase (6,10) or exhibit Stribeck-like behavior (9). For polyelectrolytes, friction increases with sliding velocity due to changes in the water layer at the interface caused by the electrostatic repulsion between the electric double layers (11). While the sliding speed was not varied in our experiments, it can be postulated that the friction coefficient of these P(AAm-*co*-AA) hydrogels would increase with sliding speed.

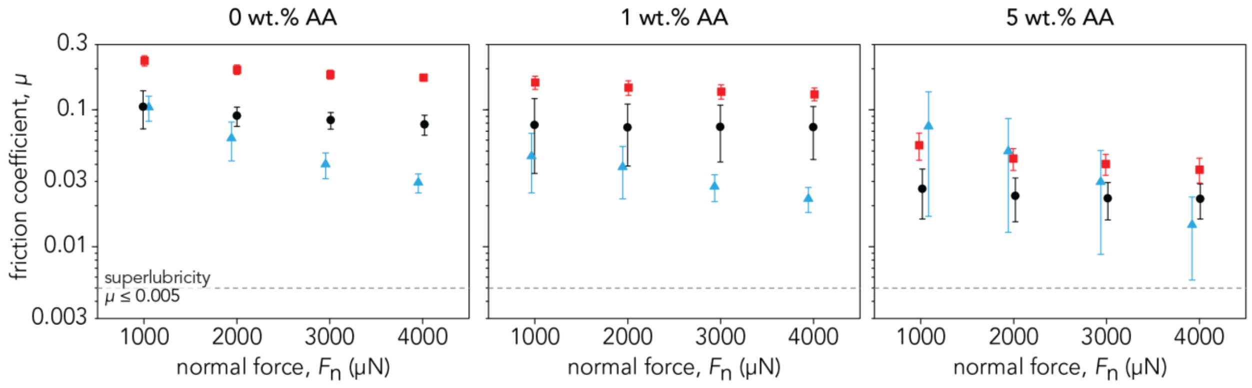

Fig S8. Friction coefficient, μ, as a function of applied normal force, F_n for P(AAm-*co*-AA) hydrogels at three AA concentrations (0, 1, and 5 wt.% AA). The effects of normal force on the friction coefficient varied depending on pH. The friction coefficient for the gels at pH = 13.8 (blue triangles) noticeably decreased with increasing force. For the gels at pH = 0.35 (red squares) and pH = 7 (black circles), the friction coefficient slightly decreased with increasing force or stayed relatively constant.

9 Relationship Between Water Content, Elastic Modulus, and Friction Coefficient

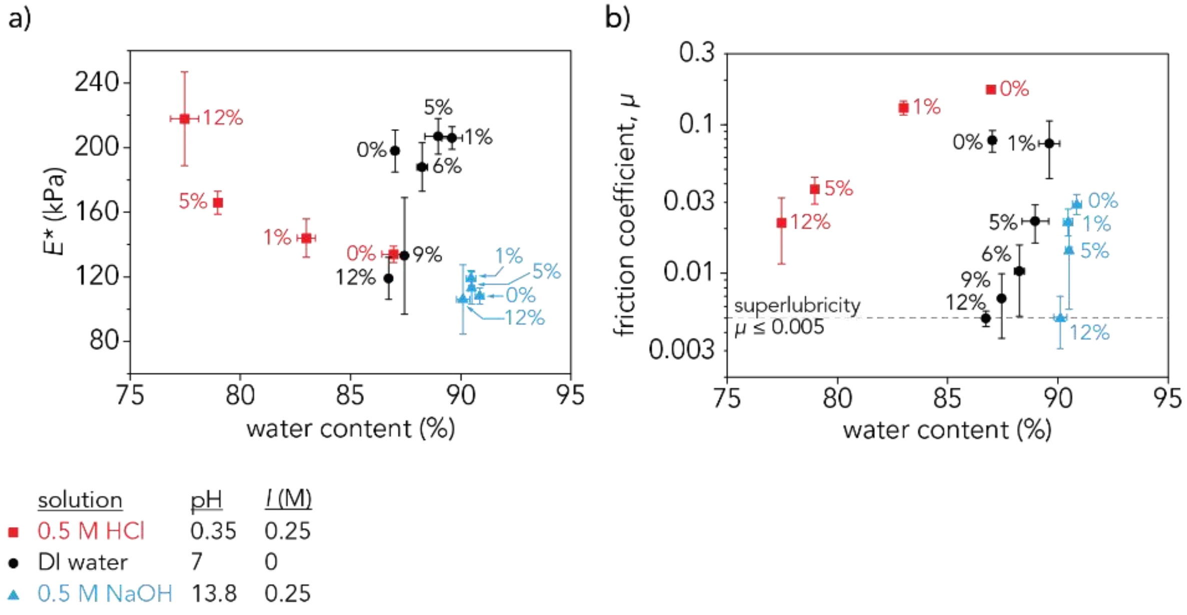

Fig S9. (a) Reduced elastic modulus, E^*, and **(b)** friction coefficient, μ, as a function of water content (%). Each data point is labeled with the AA wt.%. There is no clear trend between water content and E^* or μ. The modulus and the friction coefficient do not predictably decrease with increasing water content.

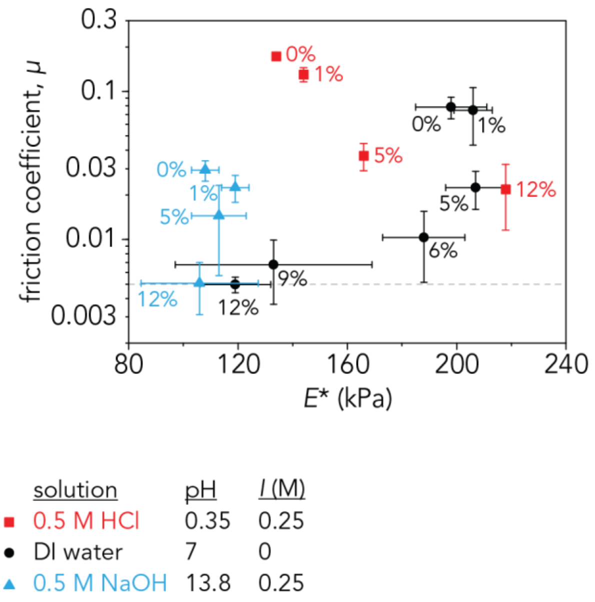

Fig S10. Friction coefficient, μ, as a function of reduced elastic modulus, E^*. Each data point is labeled with the AA wt.%. There is no clear trend between hydrogel stiffness and friction.

10 Effects of Broken Crosslinks on the Mechanical and Tribological Properties of Hydrogels

P(AAm-*co*-AA) hydrogels with 0 wt.% and 12 wt.% were characterized before and after one freeze-thaw cycle. After swelling for at least three days in DI water, the gels were characterized via microindentations and sliding experiments and then placed in -18ºC without any excess liquid for at least 12 h. The gels were then left to thaw at room temperature (23ºC) and then characterized again. This freeze-thaw cycle mechanically degraded the gels, physically breaking crosslinks to simulate the potential chemical degradation experienced by the hydrogels at pH = 13.8 through hydrolysis. At 0 wt.% AA, E^* and μ both decreased after mechanical degradation, but at 12 wt.% AA, E^* decreased while μ stayed the same. This may explain why the gels at pH = 13.8 had lower μ than the gels at pH = 7 at lower AA wt.% (0 – 5 wt.%). Any potential electrostatic interactions, or lack thereof, were trivial compared to crosslink degradation via hydrolysis.

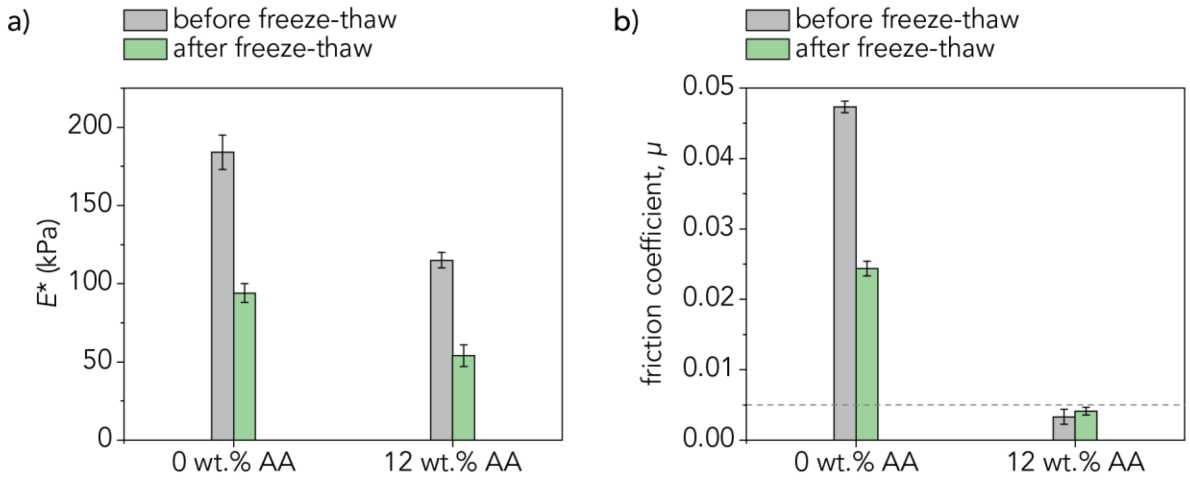

Fig S11. (a) Reduced elastic modulus, E^*, and **(b)** friction coefficient, μ, of P(AAm-*co*-AA) hydrogels at 0 wt.% and 12 wt.% before and after one freeze-thaw cycle. For P(AAm-*co*-AA)-0, E^* and μ decreased after mechanical degradation of the gel through freeze-thaw. For P(AAm-*co*-AA)-12, E^* decreased but μ stayed the same.

11 Influence of pH on Reactivity Ratios

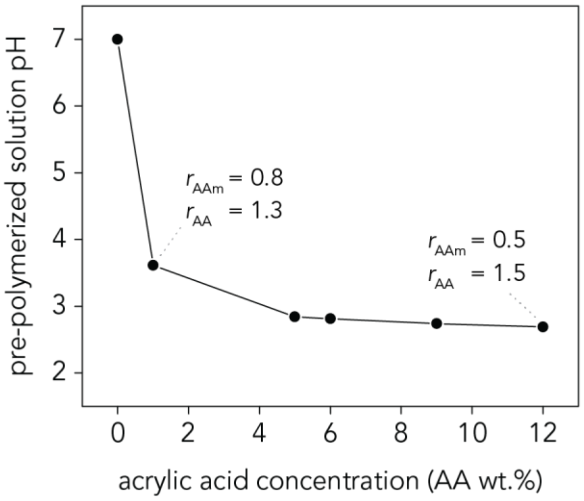

Fig S12. Pre-polymerized solution pH as a function of AA concentration labeled with reactivity ratios of AAm (r_{AAm}) and AA (r_{AA}) from Ref. (12). While there is a slight decrease in the reactivity ratio for AAm and increase for AA with increasing AA concentration, the general trend of $r_{AAm} < 1$ and $r_{AA} > 1$ stays consistent.

12 References

1. Shaw MC, Nussdorfer Jr. TJ. An Analysis of the Full-Floating Journal Bearing. National Advisory Committee for Aeronautics. 1947.

2. Hamrock BJ, Dowson D. Elastohydrodynamic Lubrication of Elliptical Contacts for Materials of Low Elastic Modulus I — Fully Flooded Conjunction. Transactions of the ASME. 1978;100:236–44.

3. de Gennes PG. Scaling Concepts in Polymer Physics. Ithaca: Cornell University Press; 1979.

4. Denisin AK, Pruitt BL. Tuning the Range of Polyacrylamide Gel Stiffness for Mechanobiology Applications. Applied Materials and Interfaces. 2016;8:21893–902.

5. Prouvé E, Drouin B, Chevallier P, Rémy M, Durrieu MC, Laroche G. Evaluating Poly(Acrylamide-co-Acrylic Acid) Hydrogels Stress Relaxation to Direct the Osteogenic Differentiation of Mesenchymal Stem Cells. Macromolecular Bioscience. 2021;21(6):1–12.

6. Gong JP, Higa M, Iwasaki Y, Katsuyama Y, Osada Y. Friction of Gels. Journal of Physical Chemistry B. 1997;101:5487–9.

7. Urueña JM, McGhee EO, Angelini TE, Dowson D, Sawyer WG, Pitenis AA. Normal Load Scaling of Friction in Gemini Hydrogels. Biotribology [Internet]. 2018;13(January):30–5. Available from: https://doi.org/10.1016/j.biotri.2018.01.002

8. Gong JP, Iwasaki Y, Osada Y. Friction of Gels. 3. Friction on Solid Surfaces. Journal of Physical Chemistry B. 1999;103:6001–6.

9. Wang Z, Li J, Liu Y, Luo J. Macroscale superlubricity achieved between zwitterionic copolymer hydrogel and sapphire in water. Materials and Design [Internet]. 2020;188:108441. Available from: https://doi.org/10.1016/j.matdes.2019.108441

10. Pitenis AA, Schulze KD, Nixon RM, Dunn AC, Krick BA, Sawyer WG, et al. Polymer fluctuation lubrication in hydrogel gemini interfaces. Soft Matter. 2014;10:8955–62.

11. Oogaki S, Kagata G, Kurokawa T, Kuroda S, Osada Y, Gong JP. Friction between like-charged hydrogels - Combined mechanisms of boundary, hydrated and elastohydrodynamic lubrication. Soft Matter. 2009;5(9):1879–87.

12. Rintoul I, Wandrey C. Polymerization of ionic monomers in polar solvents: Kinetics and mechanism of the free radical copolymerization of acrylamide/acrylic acid. Polymer (Guildf). 2005;46(13):4525–32.
